# Modeling, Simulation Methods and Characterization of Photon Detection Probability in CMOS-SPAD

**DOI:** 10.3390/s21175860

**Published:** 2021-08-31

**Authors:** Aymeric Panglosse, Philippe Martin-Gonthier, Olivier Marcelot, Cédric Virmontois, Olivier Saint-Pé, Pierre Magnan

**Affiliations:** 1 Department of Electronic Optic and Signal, ISAE-SUPAERO, University of Toulouse, 31055 Toulouse, France; philippe.martin-gonthier@isae-supaero.fr (P.M.-G.); olivier.marcelot@isae-supaero.fr (O.M.); Pierre.Magnan@isae.fr (P.M.); 2 CNES, The French National Space Agency, 31400 Toulouse, France; cedric.virmontois@cnes.fr; 3 Airbus Defence and Space, 31400 Toulouse, France; olivier.saintpe@airbus.com

**Keywords:** single photon avalanche diode (SPAD), complementary metal-oxide semiconductor (CMOS), modeling and simulations, photon detection probability (PDP), quantum efficiency (QE), technology computer-aided design (TCAD), Matlab, light detection and ranging (Lidar)

## Abstract

Single-Photon Avalanche Diodes (SPAD) in Complementary Metal-Oxide Semiconductor (CMOS) technology are potential candidates for future “Light Detection and Ranging” (Lidar) space systems. Among the SPAD performance parameters, the Photon Detection Probability (PDP) is one of the principal parameters. Indeed, this parameter is used to evaluate the SPAD sensitivity, which directly affects the laser power or the telescope diameter of space-borne Lidars. In this work, we developed a model and a simulation method to predict accurately the PDP of CMOS SPAD, based on a combination of measurements to acquire the CMOS process doping profile, Technology Computer-Aided Design (TCAD) simulations, and a Matlab routine. We compare our simulation results with a SPAD designed and processed in CMOS 180 nm technology. Our results show good agreement between PDP predictions and measurements, with a mean error around 18.5%, for wavelength between 450 and 950 nm and for a typical range of excess voltages between 15 and 30% of the breakdown voltage. Due to our SPAD architecture, the high field region is not entirely insulated from the substrate, a comparison between simulations performed with and without the substrate contribution indicates that PDP can be simulated without this latter with a moderate loss of precision, around 4.5 percentage points.

## 1. Introduction

Laser remote sensing is remodeling many fields, from Earth observation with Low-Earth Orbit (LEO) satellites to self-driving cars. The working principle is quite simple as it consists of emitting an optical pulse and analyzing the reflected and back-scattered light to probe a scene [1].

SPAD high sensitivity with low photon flux detection capabilities [2], excellent jitter or time resolution [3], and small dead time [4] make them suitable for future space-borne Lidar missions. CMOS technologies development improved these devices, with the possibility to combine the SPAD detector and its driving electronic [5] on the same integrated circuit. With this significant progress, SPAD benefits from the maturity, relatively low manufacturing cost, and dynamism of the CMOS industry. Moreover, CMOS SPAD is particularly appropriate for the space-borne Lidar wavelength of interest of 532nm due to the silicon absorption spectrum properties in the visible range.

CMOS SPAD performance prediction is crucial to optimizing SPAD design before manufacture since the CMOS industry workflow is time-consuming and costly. This paper is focused on the Photon Detection Probability (PDP) which is the probability for an incident photon to trigger an avalanche breakdown. Commercial finite element simulator tools (TCAD) accurately simulate electrical and physical parameters as the electric field or leakage current, and its outputs can be translated into SPAD metrics to compute performance parameters as the PDP. Therefore, in the last few years, multiple efforts contributed to improving the state-of-the-art in SPAD performance parameter modeling and simulations. The first attempt we have reported is from Kindt et al. [6], on a custom process, using abrupt p-n junction approximation and a mathematical formalism that will inspire the next attempts. Kang et al. [7] proposed an analytical model to describe the behavior of PDP for SPAD working in the wavelength range 1000–1500 nm, and used commercial avalanche photodiodes in InGaAs for their model validation. Gulinatti et al. [8] simulated the PDP for the wavelength range 400–1000 nm and applied their model to custom silicon SPAD structure (thin SPAD) designed in their laboratory. They employed an approach based on measured doping profile to estimate the electric field with TCAD, combined with analytical and numerical methods to calculate intermediate physical parameters, such as transmission coefficient, carriers diffusion, or avalanche triggering probabilities. A very similar structure was also investigated by Mazzillo et al. [9], using device simulations with spreading resistance profile analyses to obtain doping data. The authors have taken many assumptions, as they assumed an abrupt junction shape to perform their calculation, as well as the value of intermediate physical parameters like the breakdown voltage and the depletion layer thickness. They also extrapolated some other key elements as the diffusion length. Predictions were performed for the wavelength range 380–520 nm.

Concerning standard CMOS processes, Rochas et al. [5] performed simulation on a standard 0.8μm process, using abrupt p–n junction approximation. More recently, Xu et al. [10] and Hsieh et al. [11] proposed modeling and simulation approaches, applied to respectively 150nm and 0.8μm CMOS processes. These authors generate the processes entirely with commercial device simulator tools, which require the access to confidential foundry data to adjust the process recipes correctly. Other remarkable contributions for InGaAs/InP SPAD have been published by Knezevic et al. [12], Signorelli et al. [13], and Xie et al. [14].

In line with these previous studies, we propose a modeling and simulation method based on a combination of:

- Doping profile measurement. The main distinction with the previous approach is the use of measured doping profile from commercial standard CMOS processes applied to a structure available in such processes, instead of custom processes in [8], an abrupt p-n junction shape approximation in [5,6,9], InGaAs diodes in [7], and entirely generated processes from commercial device simulator tools in [10,11].
- A commercial device simulator tool (TCAD).
- Analytical/numerical relations computed using a Matlab routine.

Our approach philosophy is close to Gulinatti et al. [8], as they also used measured doping profiles to feed their TCAD tool, although we have differences in the model used, the implementation, and validation device. In terms of modeling, we propose an additional parameter to adjust the contribution of charges within the substrate for SPAD without complete high-field region insulation, as presented in Section 2.4. With our SPAD design presenting this particularity, we propose a comparison based on simulations performed with and without the substrate electron contribution to evaluate the significance of this latter to the PDP. We also propose the evaluation of the minority carrier lifetime with TCAD, to model the fraction of carriers that recombine in the neutral regions, as it was neglected in [8]. Concerning the ionization coefficient modeling, they used the mean free path as a fitting parameter to adjust the breakdown voltage, while we take commonly used ionization coefficients, with the addition of a temperature dependence coefficient, as presented in Section 2.6. The main differences in the implementation lie in the choice of TCAD output, presented in Section 2.7. Concerning the validation device, we designed our own SPAD in standard 180nm CMOS process to validate the method for the 450–950 nm wavelength range. Therefore, a standard CMOS process is used against a custom process with an n-type wafer and a p-type double epitaxial layer in [8]. The different technologies engender significant differences in terms of doping distribution and impacts breakdown voltage and the over-voltage, with a notable difference in the orders of magnitude.

The model aims at being generic and usable for any SPAD, regardless of its architecture (layout) or size, notably for SPAD envisioned for future space-borne Lidars. This paper is structured as follows: Section 2 describes the model and simulation method, Section 3 presents the layout of the SPAD employed for validation, and the PDP characterization method. Finally, Section 4 introduces simulation and measurement results followed by a discussion.

## 2. Model and Simulation Method Description

To be detected, the photon must make its path throughout the passivation layer on top of the silicon surface before it reaches the multiplication region of interest and triggers the breakdown. Hence, we broke down the photon detection into two phases: the photon transmission and the avalanche triggering. In the first phase, we define $P_{T}$ as the probability of photon transmission through the passivation layer. $P_{T}$ depends on the photon wavelength λ and on the dielectric characteristics. In the second phase, once the photon reaches the silicon surface, it needs to be absorbed (creating an electron-hole pair) and trigger an avalanche. The probability of occurrence of such phenomenon is called the internal quantum efficiency, noted QE. This latter is also a function of λ and PDP is [6,9,10]:

(1) $$PDP=P_{T}\times QE.$$

The following subsections describe the models used to estimate transmission $P_{T}$ and internal quantum efficiency QE.

### 2.1. Photon Transmission Modeling

**Figure 1:**
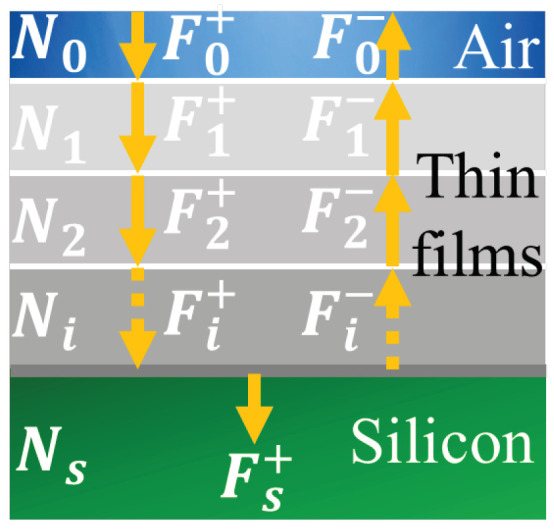
Transmission through thin films, incident and reflected electrical field.

We implemented the matrix approach [15] and the theory of optical transmission in thin-film to model the photon transmission throughout the passivation layer. As depicted in Figure 1, the electric field was separated in an incident $F_{i}^{+}$ and a reflected $F_{i}^{-}$ field for each layer *i*. The relation between layers *i* and i+1 depends on the Fresnel transmission and reflection coefficient, the phase shift $\delta_{i}$, the thickness $d_{i}$, the angle of incidence of incoming photon $\theta_{i}$, the refraction coefficient $N_{i}$, the material extinction coefficient $k_{i}$ and the material reflection coefficient $n_{i}$ of layer *i*.

Applying the matrix approach to evaluate the electric field and considering the reflection in the silicon is null $F_{s}^{-}=0$, the thin-films transmission $P_{T}$ is:

(2) $$P_{T}=\frac{n_{s}}{n_{0}}{\left|\frac{F_{S}^{+}}{F_{0}^{+}}\right|}^{2},$$

The theory predicts oscillations on the transmission as several parameters like the phase shift or the angles of incidence are modeled with trigonometric functions. Their frequencies depend upon the phase shift, and increases when the wavelength decreases and/or the layer thickness increases. Figure 12 shows these oscillations on the simulated PDP for $V_{Ex}=20\%V_{Br}$. We applied this model to our passivation layer displayed in Section 4.1. Other papers simulate transmission using a similar approach [8]. The TCAD electrical simulator that we used, Sentaurus Device, is also able to implement the 1D Transfer Matrix Method to compute the transmission [16].

### 2.2. Internal Quantum Efficiency Modeling

**Figure 2:**
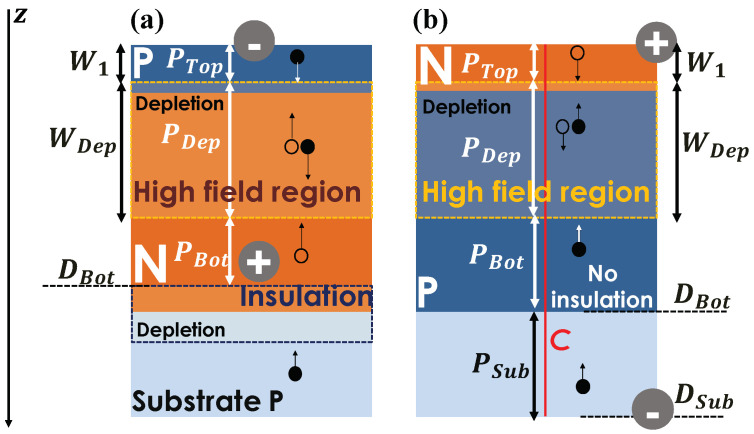
Typical SPAD cross-sections. (**a**) A SPAD with complete insulation between the high field region and (**b**) a SPAD without insulation.

The Internal Quantum Efficiency, QE, is the sum of several contributions. Figure 2 shows two typical cross-sections. For the junction on Figure 2a, the high field region is completely insulated from the substrate. Indeed, if an electron from the substrate reaches the edge of the second depletion region, at the interface between the N implant and the substrate, it will be collected at the cathode contact, far from the high field region. This configuration is found in many SPAD with different process nodes [17,18,19]. Depending on their wavelength, photons are absorbed in silicon with different penetration depths. The photon can be absorbed into three zones: the depleted region Dep and the undepleted regions, Top and Bot, respectively on the top and at the bottom, of the depleted layer Dep. To be detected, a photon within the Top region must:

- be absorbed, creating an electron–hole pair;
- the resultant particle has to diffuse to the depletion layer without recombination;
- once the particle reaches the depletion region, it needs to be accelerated by the high electric field, and finally, triggers an avalanche.

$P_{Top}$ is the probability for this succession of events to occur. Similarly, $P_{Bot}$ is the symmetrical probability for the bottom region. $P_{Dep}$ is the probability that either an electron or a hole, generated at position *z* within the depleted region, triggers an avalanche. For the junction on Figure 2b, the high-field region is not entirely insulated from the substrate and charges generated within the substrate can diffuse to the depletion region and trigger an avalanche. These charges have to cross the potential barrier between P-well and substrate. To model this contribution, we introduce the probability $P_{Sub}$. Then, the internal quantum efficiency QE becomes:

(3) $$QE=P_{Top}+P_{Dep}+P_{Bot}+P_{Sub}$$

where QE depends on λ. This equation can be simplified if the high electric field area is insulated from the substrate with $P_{Sub}=0$.

The section below gives details about each contribution.

### 2.3. Internal Quantum Efficiency Modeling in the Undepleted Regions

The mathematical formalism of Section 2.3, Section 2.4 and Section 2.5 is from [6,9]. The modeling method is given for the case represented Figure 2b. QE and the electrical and statistical parameters are calculated along the *C* axis in Figure 2b.

Our SPAD was circular and exhibits a rotational symmetry, hence the *C* axis represents a typical cross-section of the photosensitive area of the sensor.

Within the first region corresponding to the N undepleted layer, photons have to be absorbed, and then the generated hole must diffuse to the upper limit $W_{1}$ of the depleted area. Let $\alpha_{Abs}$ be the photon absorption coefficient at wavelength λ in silicon. The photon absorption probability with the generation of an electron-hole pair at the depth *z* is:

(4) $$P_{Abs}=\alpha_{Abs}e^{-\alpha_{Abs}z}.$$

The probability for a hole to reach the upper limit $W_{1}$ without Auger or Shockley-Read-Hall recombination is:

(5) $$P_{h_{diff}}=e^{-\left(\frac{W_{1}-z}{L_{h}}\right)},$$

with $L_{h}$ the hole diffusion length inside the undepleted N region. Diffusion length $L_{e,h}$, respectively for electrons and holes, depends on the diffusion coefficient $D_{e,h}$ and on the carriers lifetime $\tau_{e,h}$:

(6) $$L_{e,h}=\sqrt{D_{e,h}\times \tau_{e,h}}.$$

The diffusion coefficient $D_{e,h}$ depends on carriers mobility $\mu_{e,h}$, temperature *T*, the elementary charge *q* and the Boltzmann constant *k*:

(7) $$D_{e,h}=\mu_{e,h}\times \frac{kT}{q}.$$

We define $P_{1}$ as the probability that a photon transmitted through the entrance interface is absorbed in the undepleted N region and the corresponding hole reaches the depletion layer upper limit at $z=W_{1}$ without Auger or Shockley–Read–Hall recombination:

(8) $$P_{1}=\int_{0}^{W_{1}}\alpha_{Abs}e^{-\alpha_{Abs}z}e^{-\frac{W_{1}-z}{L_{h}}}dz.$$

Once the hole arrives in the depletion region, it must drift inside the high electron field and trigger an avalanche, consequently:

(9) $$P_{Top}=P_{1}P_{ph}\left(W_{1}\right).$$

$P_{ph}\left(z\right)$ is the probability for a hole starting from position *z* in the depleted region to trigger an avalanche breakdown. The modeling of this probability is given in Section 2.6.

Similarly, we obtain $P_{Bot}$ in the undepleted P region. We define $P_{2}$ as the probability that a photon transmitted is absorbed in the undepleted P region and the corresponding electron diffuses to the bottom limit of the undepleted region without recombination:

(10) $$P_{2}=\int_{W_{1}+W_{Dep}}^{D_{BOT}}\alpha_{Abs}e^{-\alpha_{Abs}z}e^{-\frac{z-\left(W_{1}+W_{Dep}\right)}{L_{e}}}dz.$$

With $D_{BOT}$ the z-coordinate of the interface between the undepleted P region and substrate. The internal quantum efficiency of undepleted P region is:

(11) $$P_{Bot}=P_{2}P_{pe}\left(W_{1}+W_{Dep}\right),$$

with $P_{pe}\left(z\right)$ the symmetric of $P_{ph}\left(z\right)$, for electrons.

### 2.4. Internal Quantum Efficiency Modeling in the Substrate

An additional contribution has to be taken into account when the high field region is not entirely insulated from the substrate, which is the case of Figure 2b. Charges generated within the substrate or charges from the surrounding electronics can diffuse to the depletion layer. Although, not all the charges from the substrate are collected, for instance, they may have to cross the potential barrier between P-well and substrate. As a consequence, the contribution from the substrate $P_{Sub}$ will be similar to $P_{Bot}$, with a fitting parameter $P_{\Delta_{e}}$ to adjust the number of charges collected from the substrate. Thus:

(12) $$P_{Sub}=P_{\Delta_{e}}P_{3}P_{pe}\left(W_{1}+W_{Dep}\right),$$

with:

(13) $$P_{3}=\int_{D_{Bot}}^{D_{Sub}}\alpha_{Abs}e^{-\alpha_{Abs}z}e^{-\frac{z-\left(W_{1}+W_{Dep}\right)}{L_{e}}}dz,$$

with $D_{Sub}$ the depth of the substrate.

The introduction of the parameter $P_{\Delta_{e}}$ and the influence of the substrate contribution will be discussed in Section 4.

### 2.5. Internal Quantum Efficiency Modeling in the Depleted Region

Inside the depleted region, a photon is absorbed and generates an electron-hole pair with the probability $P_{Abs}$. This pair will generate an avalanche breakdown with the probability $P_{p}$ detailed in Section 2.6. Then, the internal quantum efficiency in the depleted region is:

(14) $$P_{Dep}=\int_{W_{1}}^{W_{1}+W_{Dep}}\alpha_{Abs}e^{-\alpha_{Abs}z}P_{p}\left(z\right)dz.$$

### 2.6. Avalanche Triggering Probability Modeling

The avalanche breakdown probability is an essential parameter used for each performance parameter calculation. The TCAD tool available to us does not implement its calculation, consequently we use a Matlab routine. The relations describing avalanche triggering depend on the chosen model. In this paper, we employ the Oldham and McIntyre local model [20,21] with a common set of ionization coefficients [22]. We chose this set of coefficients as it allows a breakdown voltage evaluation in good agreement with the measurements made on our structure, as presented in Section 4.2.

$P_{p}\left(z\right)$ is the probability that either an electron or a hole, starting from the position *z* inside the depletion region, triggers an avalanche breakdown:

(15) $$P_{p}\left(z\right)=P_{pe}\left(z\right)+P_{ph}\left(z\right)-P_{pe}\left(z\right)P_{ph}\left(z\right),$$

with $P_{pe}\left(z\right)$ and $P_{ph}\left(z\right)$ the probability for respectively an electron and a hole, starting from the position *z* inside the depletion region to trigger an avalanche. In the local model, these probabilities are related to the following system of equations:

(16) $$\frac{dP_{pe}}{dz}=\left(1-P_{pe}\right)\alpha_{e}\left(P_{pe}+P_{ph}-P_{pe}P_{ph}\right)$$

(17) $$\frac{dP_{ph}}{dz}=-\left(1-P_{ph}\right)\alpha_{h}\left(P_{pe}+P_{ph}-P_{pe}P_{ph}\right)$$

$\alpha_{e}$ and $\alpha_{h}$ are, respectively, the ionization coefficient for electrons and holes. We include a temperature dependence in the ionization coefficients according to the method proposed by [16].

### 2.7. Model Implementation

**Figure 3:**
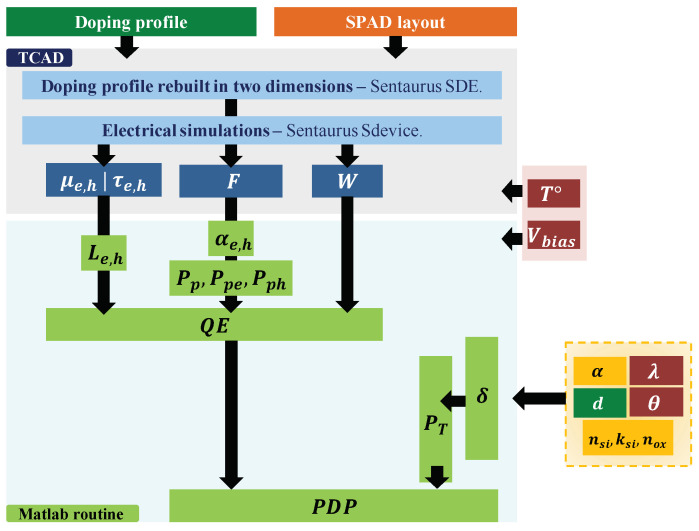
Photon Detection Probability (PDP) modeling implementation, based on TCAD, Matlab routine, measurements and external data.

Figure 3 shows the Photon Detection Probability PDP modeling implementation.

To acquire the doping profile, the measurement technique used is the Secondary Ion Mass Spectrometry (SIMS measurement) performed on a dedicated structure [23]. These measurements were executed on structures that present dedicated areas made specifically with each implant to characterize. These areas are boxes with sides around 100–200 μm. This method introduces some uncertainties on the doping profile which could lead to potential TCAD errors, as the software would be fed by these measurements. Indeed, as the measurements are performed on each implant individually, some complex interaction effects could not be modeled by the approach. These interactions could depend on multiple factors such as the implant, annealing or the species involved. Once the doping profile of each implant is obtained, it is injected in the TCAD tool to rebuild the SPAD in two dimensions.

The transmission probability $P_{T}$ is evaluated with the Matlab routine, using the following inputs: the photon wavelength λ, the angle of incidence of incoming photon θ, the thickness of each material layer *d*, the silicon and oxide material refractive index $n_{si}$, $n_{ox}$, and the silicon extinction coefficient $k_{si}$.

The PDP calculation is executed alongside the vertical axis *C* showed in Figure 2. Evaluated alongside this axis, the TCAD electrical device simulation provides:

- carriers mobility $\mu_{e,h}$, modeled with the Masetti model [16,24] for modeling mobility degradation due to impurity scattering. The Philips unified mobility model, proposed by Klaassen [25], is also used. This latter unifies the description of majority and minority carrier bulk mobilities, and takes into account the temperature, electron-hole scattering, screening of ionized impurities by charge carriers and clustering of impurities. The high-field saturation is also taken into account [16];
- carrier lifetimes $\tau_{e,h}$, modeled with doping, electric field and temperature dependence [16]. Carrier lifetimes are defined by the predominant carrier recombination-generation mechanisms: the band-band phonon-assisted Auger-impact and the capture-emission Shockley–Read–Hall (SRH) process. Lifetimes are then controlled by the densities of defects in the silicon [26]. Nevertheless, a doping dependence of the lifetimes are experimentally observed in silicon technologies and is modeled by the Scharfetter relation. This latter is based on theoretical conclusion that trap density of defects obtained with ionic implantation is strongly related to the doping density [26]. More details about the minority carriers lifetime implementation are given in our previous paper [27];
- the electric field *F* and the depletion layer width $W_{Dep}$, obtained by Poisson equation resolution starting from the doping profile [16].

These electrical data are then injected in the Matlab routine. The program evaluates the diffusion length $L_{e,h}$, the ionization coefficients $\alpha_{e,h}$, and the avalanche triggering probabilities $P_{p}$, $P_{pe}$ and $P_{ph}$. Environment data such as the temperature *T*, the bias voltage $V_{bias}$, complete the model implementation. In our model, we defined the excess voltage as $V_{Ex}=V_{bias}-V_{Br}$, with $V_{Br}$ the breakdown voltage. To finalize the Quantum Efficiency calculation, we use the wavelength λ, which determines the absorption coefficient $\alpha_{Abs}$.

For the purposes of this study it is assumed that PDP does not substantially change with device area and that understanding the central PDP is meaningful.

Feasibility studies performed by Airbus DS suggest that large SPAD should be used for their future projects of spaceborne Lidars: between 100 and 200 μm in diameter, or a configuration similar to a Silicon Photomultiplier (SiPM) with 2 × 2 or 4 × 4 SPAD sensors, with a respective diameter of 50 or 25 μm. Indeed, the detector size is dimensioned by the optical interface. The light beam coming from the optical system located above the detector is widely opened, around f/1, and it is not possible to target optical spot sizes, comprising all the return echo energy, lower than 150–200 μm.

Provided the SPAD design allows us to avoid phenomena such as optical diffraction effects for very small active areas and/or guard rings merge leading to a lower electric field and/or higher breakdown voltage, the model should be applicable to simulate the various set of SPAD dimensions envisioned for spaceborne Lidar. Further measurements will have to be performed on various SPAD sizes, to confirm this statement. However, other works confirm this trend, as in [8], where the authors verified experimentally that no difference was observed for PDP for various SPAD dimensions, coming from the same lot.

Once the simulations are completed, we compared the simulated and measured PDP to validate the model, as presented in Section 4.3.

## 3. SPAD Description and Characterization Method

**Figure 4:**
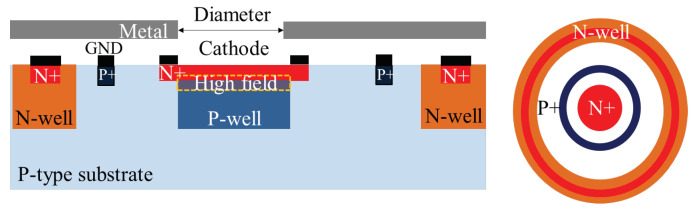
Cross-section and top view of the SPAD under investigation.

**Figure 5:**
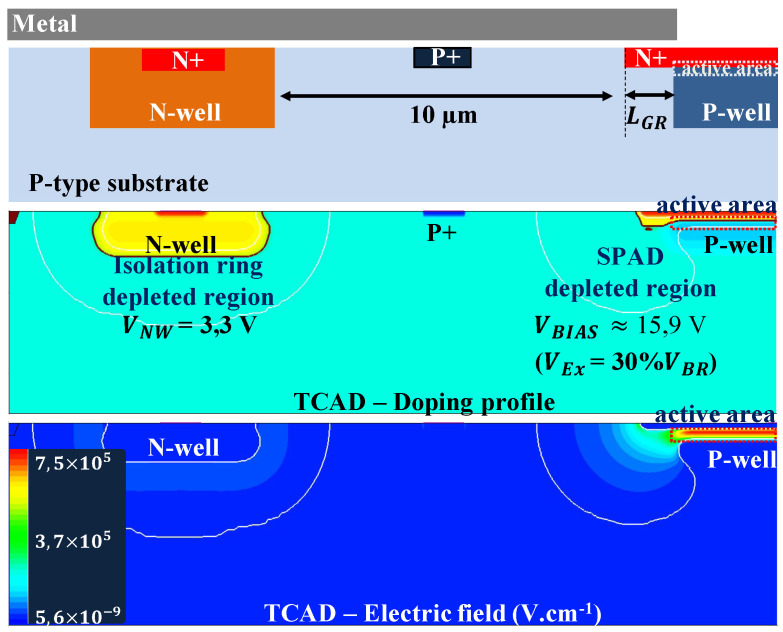
2D electrical field distribution of the SPAD under investigation for an excess voltage $V_{Ex}$ = 30% $V_{Br}$). The plots “TCAD–Doping profile” and “TCAD–Electric field” are directly taken from the TCAD software and are drawn to scale.

We developed a CMOS SPAD in 180nm CMOS process for the model validation. To set the architecture, we performed several TCAD simulations using the standard CMOS processes available in our library. Then, we selected the combination of CMOS process and architecture that avoid the premature edge breakdown (PEB). A cross-section of the SPAD is illustrated Figure 4 and the simulated 2D electrical field distribution is represented in Figure 5.

The high electric field is related to the junction between the N+ implant and the P-well. This region must be photosensitive as it is the active area. With the doping profile measurements and TCAD simulations we performed, no implant were available to us to insulate the high field region without creating a PEB or other issues. In consequence, the main drawback of our structure lies in the incomplete high field region insulation from the substrate. To lower the number of parasitic charges captured by the high electric field, we add an N-well insulation ring. This insulation method is not perfect, as only the charges passing in its neighborhood are captured and deeper parasitic substrate charges can diffuse to the high electric field. Metal layers are used to shield the non-photosensitive areas and enable the reduction of the number of photo-generated charges inside the substrate.

Our SPAD has a circular shape of 10μm diameter. We performed TCAD simulation to size our SPAD avoiding the punch-through phenomenon and premature edge breakdown (PEB). In general, SPAD are operated with excess voltages between 10 and 30% of the breakdown voltage value. To ensure the proper functioning for bias voltage in this range, we make sure:

- to avoid premature edge breakdown;
- to avoid diode and insulation ring depletion layer merging.

The driving electronic to quench the avalanche and shape the signal is composed of a resistor (passive quenching) and a buffer co-integrated with the SPAD.

The PDP measurements are achieved with excess voltages between 15 and 30% of the breakdown voltage. We illuminate the SPAD in flat field with a wide-band light source with halogen-tungsten lamp, coupled with an integrating sphere Gooch and Housego—OL Series 462. Interference filters enable the wavelength selection, between 450 and 950nm, with a 50nm step. As a result, step precision does not enable the observation of the oscillations described in Section 2.1. The filters have a precision of +/−2nm on the selected wavelength, and a full width half maximum (FWHM) of 10nm on the light intensity. These features are taken into account when applying the model. Irradiance $I_{rad}$ was measured on a dedicated reference diode, and we obtain the photon flux flux(λ) on the SPAD surface, for a given wavelength. The measured PDP is:

(18) $$PDP\left(\lambda \right)=\frac{C(\lambda )-DCR}{S_{SPAD}\times flux\left(\lambda \right)},$$

with, C(λ) the count rate of the illuminated SPAD, DCR the measured Dark Count Rate, and $S_{SPAD}$ the SPAD surface area. The photon flux is obtained with the irradiance measurements, $flux=\frac{I_{rad}}{E_{ph}}$, with $E_{ph}$ the photon energy.

We performed around 3000 oscilloscope acquisitions of 1 ms each, to obtain the DCR and C(λ). The count rate is evaluated with the mean count μ for this duration.

The dark count rate and afterpulses previously measured for the detector are removed from C(λ) to know the real count rate due to incident photons. Even if the DCR is quite high, the measurement is possible provided the SPAD does not reach the count saturation in illumination. This is achievable as the dead-time of the SPAD is around 70 ns, so provided the photon flux is not too high, the count is still possible. The complete set of performance parameters for our SPAD was measured and the results are presented in Table 1 for an excess bias voltage of 2.5 V ($V_{Ex}$ = 20% $V_{Br}$). The measurements are consistent with the literature [28,29,30,31,32,33], for CMOS 180nm process. Nevertheless, as the architectures of the SPAD are not the same and the doping profile of the CMOS processes could be different for equivalent implants, and as we did not find studies with comparable SPAD architectures made with 180 nm standard CMOS process, a direct comparison is not possible. Our DCR is quite high, at $V_{Ex}$ = 20% $V_{Br}$, our SPAD exhibits an excellent PDP of 40% at 450nm, and greater than 30% around 532nm (the expected wavelength for space borne Lidar application).

## 4. Simulation, Measurement Results and Discussion

### 4.1. Photon Transmission Simulation

For this study, the passivation layer thickness provided by the foundry is used. These layers consist of:

- silicon nitride ${\mathrm{Si}}_{3}N_{4}$ of 1.18μm thickness;
- silicon dioxide $\mathrm{Si}O_{2}$ of 3.88μm thickness. In reality, this layer is not unified and contains several $\mathrm{Si}O_{2}$ layers called Inter-Layer-Dielectric (ILD). The stacking leads to a very slight index variation that we neglect.

### 4.2. Internal Quantum Efficiency and Photon Detection Probability Simulations

In the following sections, the excess voltage $V_{Ex}$ is expressed as a proportion of the breakdown voltage. Breakdown voltage measurements are performed on 20 identical SPAD of 10μm diameter. The measured mean breakdown voltage is 12.55V with a standard deviation σ=0.1V. The TCAD simulation gives a breakdown voltage of 12.23V at ambient temperature, using a common set of ionization coefficients [22].

The measured breakdown voltage of 12.55V is used as reference for the measurements, and the simulated value of 12.23V for the simulations.

The model was applied on the cross-section presented Figure 2b. Relations described in Section 2 are applied alongside the axis *C*.

**Figure 6:**
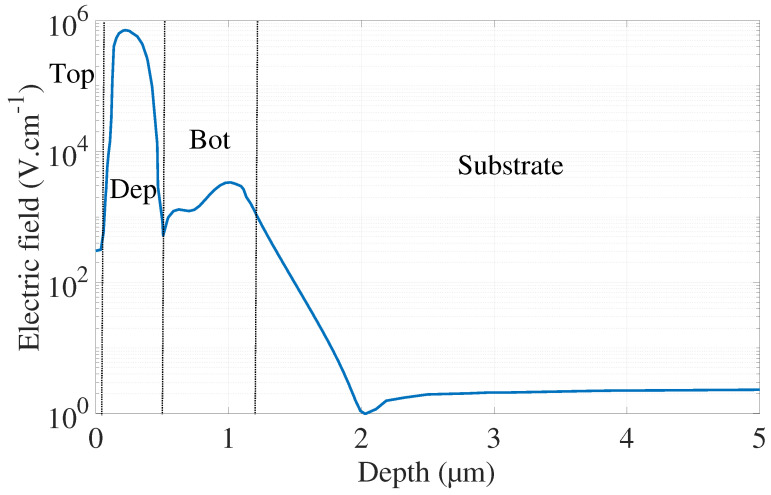
Electric field profile obtained with TCAD simulation based on doping profile measurement for $V_{Ex}=20\%V_{Br}$. A logarithmic scale is used for the vertical axis.

**Figure 7:**
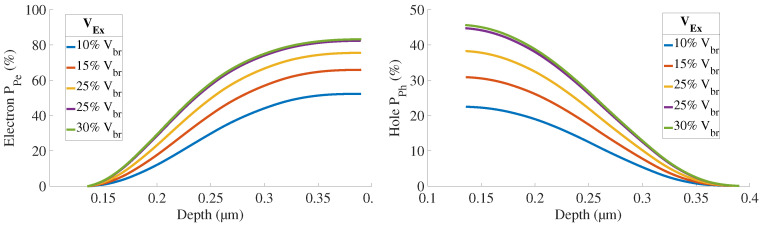
$P_{pe}\left(z\right)$ and $P_{ph}\left(z\right)$ are respectively the probability for an electron/a hole starting from the position *z* inside the depletion region to trigger an avalanche.

**Figure 8:**
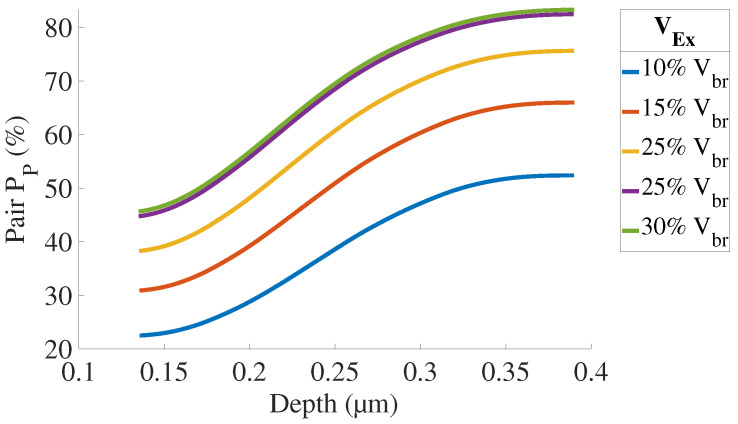
$P_{p}\left(z\right)$ is the probability that either an electron or a hole, starting from the position *z* inside the depletion region, triggers an avalanche breakdown.

The electric field is illustrated in Figure 6 for $V_{Ex}=20\%V_{Br}$ and the avalanche breakdown probabilities in Figure 7 and Figure 8.

**Figure 9:**
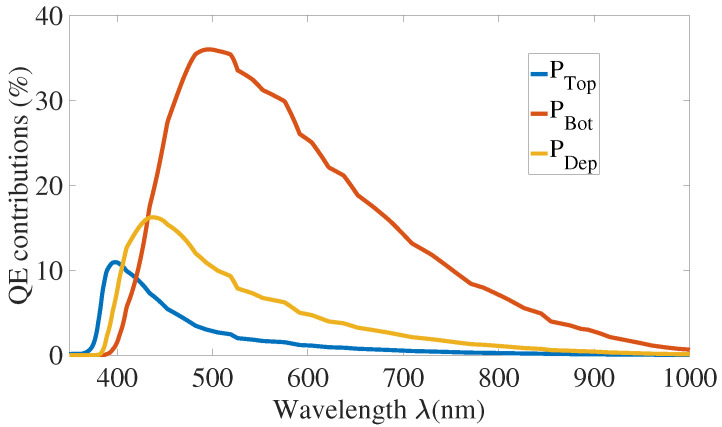
Internal quantum efficiency contributions for $V_{Ex}=20\%V_{Br}$.

Figure 9 presents the simulated internal quantum efficiency of each zone describes in Figure 2b. The contribution of the substrate will be discussed below. The undepleted Bot region shows the highest quantum efficiency. This result is coherent since:

- this zone is longer than Top and Dep regions and more charges can diffuse to the depleted region to trigger an avalanche;
- the electrons generate the breakdown in this region. Electrons have a better mobility and avalanche triggering probability than holes.

Moreover, Figure 6 highlight the relevance of introducing the $P_{\Delta_{e}}$ parameter. Indeed, the electric field drastically drops within the substrate. Therefore, electrons generated in this zone will go in more unpredictable and multiple directions than the ones generated in Bot regions. In the latter layer, electrons will benefit from the electric field and will be drifted toward the depletion region.

The potential barrier between Bot and substrate regions, make the task more difficult for electrons to reach the depleted region. As a consequence, only a small fraction of electrons generated within the substrate, modeled by the $P_{\Delta_{e}}$ parameter, will be able to make the journey until the depletion region and triggers an avalanche.

**Figure 10:**
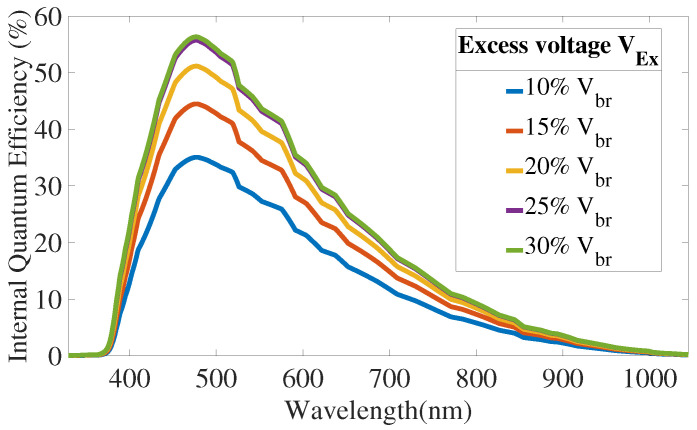
Internal Quantum Efficiency simulation results, without substrate contribution.

**Figure 11:**
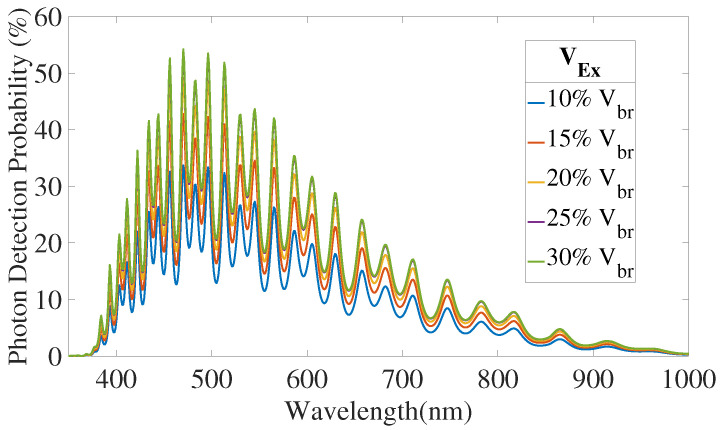
Photon Detection Probability simulation results, without substrate contribution.

Figure 10 and Figure 11 present QE and PDP simulations results.

### 4.3. Comparison with Measurements and Discussion

PDP exhibits oscillations predicted by the thin-film theory presented Section 2.1. These oscillations are not apparent in the measurements due to the step precision of the wavelength of our measurement setup. As specified in Section 3, the light intensity has a FWHM of 10nm and the simulated PDP is filtered accordingly. We assessed the mean PDP over 10nm of each measured wavelength, and called this signal “filtered PDP”. The results of our measurements are compared with this filtered signal.

Table 2 presents the mean error between measured and simulated-filtered PDP for excess voltage going from 15 to 30% of $V_{Br}$. The mean error is the average of the relative error for each wavelength, before averaging we take the absolute value of the error. These simulations were performed without the substrate contribution $P_{Sub}$. The total mean error for the range of simulated excess voltage is around 18.5%. The largest simulated-measured PDP difference occurs at 450nm, we did not identify any particular reason, but it could be due to quantum yield for high energy photons or lifetime/mobility errors for the surface region.

As presented in Section 2.4 a fraction of charges from the substrate can be collected by crossing the potential barrier between P-well and substrate. We use the fitting parameter $P_{\Delta_{e}}$ to adjust this fraction. The fitting parameter was chosen to minimize the mean error. The Table 2 summarizes also the mean error between measured and simulated-filtered PDP, when the substrate contribution is taken into account. The total mean error is 14% which makes a difference of 4.5 percentage points with the simulation performed without the substrate contribution. The decline of the fitting parameter with the raise of the excess voltage, from 15% at $V_{Ex}=15\%\;V_{Br}$ to 5% at $V_{Ex}=30\%\;V_{Br}$, suggest that a smaller fraction of charges can overcome the barrier between P-well and substrate. This indicates that fewer charges can get through the potential barrier with the raise of the bias voltage.

The PDP can be simulated without the substrate contribution with a mean error of 18.5% and the introduction of a fitting parameter to adjust the substrate contribution improves the precision around 4.5%. This result shows that the PDP can be simulated without the substrate contribution, with a moderate loss of precision, as only a small fraction of charges from the substrate, from 5% to 15% depending on the bias voltage, can cross the potential barrier between P-well and substrate.

As the use of the fitting parameter would make the model specific to a given type of SPAD, the simulation without the substrate contribution can be used as a predictive tool to simulate PDP for a broad range of excess voltage, with a moderate loss of precision.

**Figure 12:**
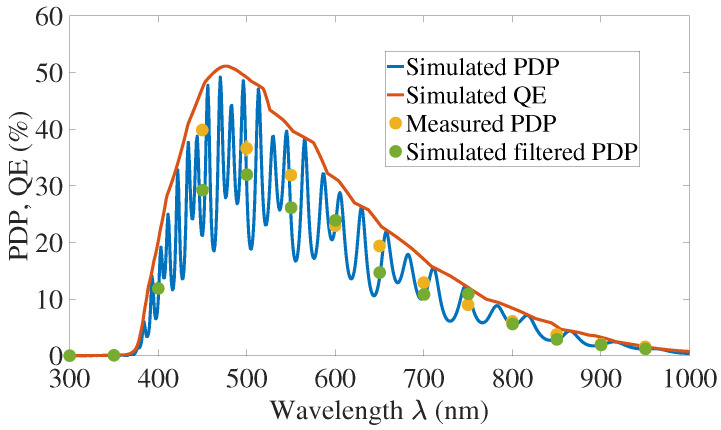
Photon Detection Probability and internal Quantum Efficiency for $V_{Ex}=20\%V_{Br}$, without substrate contribution.

**Figure 13:**
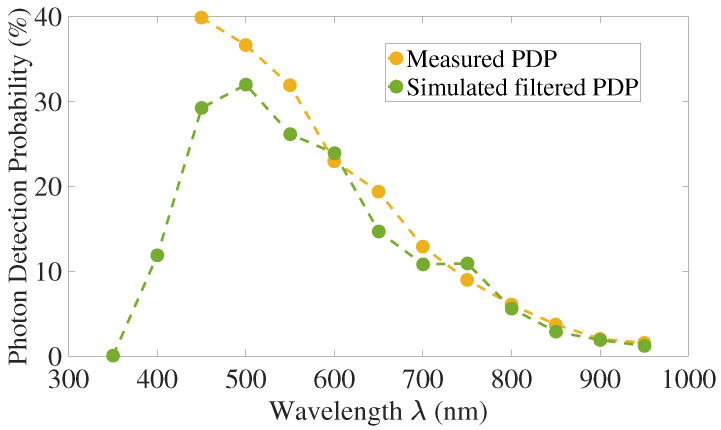
Photon Detection Probability for $V_{Ex}=20\%V_{Br}$, without substrate contribution.

Figure 12 and Figure 13 depict the results for an excess voltage of 20% of the breakdown voltage, without the substrate contribution.

On the whole, we exhibit good predictions, especially for excess voltages greater than 20% of the breakdown voltage, which are the bias condition values usually used for SPAD.

**Figure 14:**
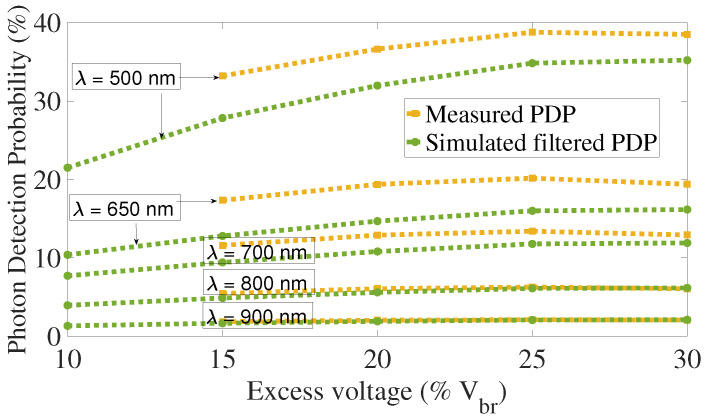
Photon Detection Probability as a function of excess voltage, without subtrate contribution.

Figure 14 shows PDP as a function of excess voltage, simulated and measured PDP are in good agreement, specially for wavelength greater than 550nm.

For the last data point, the measured PDP goes down while the excess voltage increases over 25%, we did not identify any reason for this as it should slightly increase as the simulated one. However, this decrease is small and could be related to slight errors in the measurements.

### 4.4. Uncertainties and Model Limitation

Other uncertainties can influence the characterization results:

- the FWHM of the light intensity received by the sensor, presented in Section 3;
- the slight dispersion on the breakdown voltage measurement discussed in Section 4.3.

Uncertainties can also affect the simulation:

- doping profiles measurement;
- the Inter-Layer-Dielectric (ILD) of silicon dioxide $\mathrm{Si}O_{2}$ stack has been neglected;
- incidence angle used for simulations is $0^{\circ }$, which could not be the case during measurements, although all the necessary precautions were taken, with the light source facing the SPAD.

These uncertainties are identified and remain relatively small, but provide a first indication for the mean error.

The local model was chosen, with a specific set of ionization coefficient, based on the excellent correlation between voltage breakdown measurements and simulation results. Although, it may not be the case for all CMOS processes and non-local model investigation could also be an alternative when breakdown voltages diverge too much.

## 5. Conclusions

In this paper, we develop a model to predict CMOS SPAD Photon Detection Probability PDP accurately.

We designed our SPAD to validate the model predictions. Particular attention was given to reduce parasitic capacitances, reduce recharge time, and avoid premature edge breakdown PEB, to enable PDP measurements.

A comparison between the simulation results indicates that the PDP can be simulated without the substrate contribution with a moderate loss of precision. We demonstrate a mean error of 18.5%, without the substrate contribution, for a typical range of excess voltages between 15% and 30% of the breakdown voltage.

We also have some identified uncertainties related to the thin-film on top of the silicon, doping profile, or even implant depth, which are indications to explain the mean error.

The local model for ionization coefficients is used in our study according to the good correlation between our breakdown voltage measurements and simulations.

We think that this work can help future designers to improve the conception method of future SPAD as factors important to PDP are identified and modeled.

## Figures and Tables

**Table 1:** SPAD performance parameters measured with an excess bias voltage of 2.5 V ($V_{Ex}$ = 20% $V_{Br}$).

Diameter (μm)	10
Breakdown Voltage $V_{Br}$ (V)	12.5
Excess voltage $V_{Ex}$ (V)	2.5 (20% $V_{Br}$)
Maximum PDP (%)	40 (at 450nm)
DCR (cps · $\;\mu m^{-2}$)	1700
Afterpulsing probability (%)	≤8
Jitter (ps)	260 (at 532nm)
Quenching	Passive
Dead-time (ns)	70

**Table 2:** Mean error between measured and simulated-filtered PDP for simulations performed with and without the substrate contribution $P_{Sub}$.

Excess Voltage $V_{\mathrm{Ex}}$ (% $V_{\mathrm{Br}}$)	Mean Error, without Substrate Contribution (%)	$P_{\Delta_{e}}$ (%)	Mean Error, with Substrate Contribution (%)
15	23	15	15
20	20	15	15
25	16	10	13
30	15	5	13
Average	18.5	13.75	14

## Data Availability

Not applicable.
